# Averaged Differential Expression for the Discovery of Biomarkers in the Blood of Patients with Prostate Cancer

**DOI:** 10.1371/journal.pone.0034875

**Published:** 2012-04-06

**Authors:** V. Uma Bai, Ok Hwang, George W. Divine, Evelyn R. Barrack, Mani Menon, G. Prem-Veer Reddy, Clara Hwang

**Affiliations:** 1 Department of Urology, Henry Ford Health Systems, Detroit, Michigan, United States of America; 2 Vattikuti Institute of Urology, Henry Ford Health Systems, Detroit, Michigan, United States of America; 3 Department of Internal Medicine, Henry Ford Health Systems, Detroit, Michigan, United States of America; 4 Department of Public Health Sciences, Henry Ford Health Systems, Detroit, Michigan, United States of America; 5 Josephine Ford Cancer Center, Henry Ford Health Systems, Detroit, Michigan, United States of America; The University of Kansas Medical Center, United States of America

## Abstract

**Background:**

The identification of a blood-based diagnostic marker is a goal in many areas of medicine, including the early diagnosis of prostate cancer. We describe the use of averaged differential display as an efficient mechanism for biomarker discovery in whole blood RNA. The process of averaging reduces the problem of clinical heterogeneity while simultaneously minimizing sample handling.

**Methodology/Principal Findings:**

RNA was isolated from the blood of prostate cancer patients and healthy controls. Samples were pooled and subjected to the averaged differential display process. Transcripts present at different levels between patients and controls were purified and sequenced for identification. Transcript levels in the blood of prostate cancer patients and controls were verified by quantitative RT-PCR. Means were compared using a t-test and a receiver-operating curve was generated. The Ring finger protein 19A (RNF19A) transcript was identified as having higher levels in prostate cancer patients compared to healthy men through the averaged differential display process. Quantitative RT-PCR analysis confirmed a more than 2-fold higher level of RNF19A mRNA levels in the blood of patients with prostate cancer than in healthy controls (p = 0.0066). The accuracy of distinguishing cancer patients from healthy men using RNF19A mRNA levels in blood as determined by the area under the receiving operator curve was 0.727.

**Conclusions/Significance:**

Averaged differential display offers a simplified approach for the comprehensive screening of body fluids, such as blood, to identify biomarkers in patients with prostate cancer. Furthermore, this proof-of-concept study warrants further analysis of RNF19A as a clinically relevant biomarker for prostate cancer detection.

## Introduction

While metastatic prostate cancer remains an incurable disease [1], intervention when the disease is still localized, and usually asymptomatic, is often curative [2], [3], [4]. Since early intervention is known to reduce mortality in men with prostate cancer [5], early detection holds the promise of reducing prostate cancer-specific mortality rates but its effectiveness has yet to be established. Prostate-specific antigen (PSA) is the only biomarker commonly in use for this purpose but has limitations with imperfect specificity and sensitivity. A normal PSA does not exclude the presence of potentially lethal prostate cancer [6] while benign prostatic disease can cause elevations in PSA that may require prostate biopsy for diagnosis. The limitations of PSA screening in reducing prostate-cancer mortality were seen clearly when two large randomized controlled trials published conflicting results [7], [8]. Even if interpreted in a favorable light, the results suggested that survival benefit from PSA screening was small, with 50 men needing to undergo prostatectomy to save one life [7]. This result also highlights the problem of prostate cancer overdiagnosis, when many men are diagnosed with an indolent prostate cancer that will not impact their mortality even if left untreated. However, the fact that prostate cancer remains the second-leading cause of cancer-related death in men [9] is a reminder of the importance of detecting potentially lethal prostate cancer when it is still curable. Thus, the identification of additional biomarkers to improve on the performance of PSA remains an important goal in prostate cancer early detection, especially in the detection of aggressive disease.

We describe here the usefulness of an averaged differential expression (ADE) approach to identify biomarkers in blood samples of men with prostate cancer. Differential display methodology does not require prior knowledge of RNA transcripts. Differential display thus offers an “open” system in which both known and unknown RNA transcripts associated with a disease state can be identified, a fact that distinguishes it from microarray technology [10], [11]. Differential display requires small amounts of starting RNA (as little as 20 ng total RNA), and offers an unparalleled potential for rapid identification of RNA transcripts present at different levels in test versus reference samples; it is especially efficient at identifying low abundance transcripts [11] that cannot be detected by hybridization-based microarray screening technologies. Differential display also offers an excellent opportunity to interrogate the entire transcriptome. Based on theoretical calculations, 240 pairs of arbitrary and anchor primers are expected to amplify ∼96% of expressed RNA transcripts [12]. Next-generation transcriptome sequencing (e.g., RNA-seq) shares some of these strengths (whole transcriptome analysis, prior knowledge of transcript sequence not required, small amounts of starting RNA required), while also offering the ability to distinguish allelic and splice variants that are not easily evaluated with differential display [13]. However, RNA-seq sequencing data will skew towards high abundance transcripts and the technology remains up to 100-fold more expensive than differential display [13].

ADE retains the advantages of the differential display technique while facilitating the analysis of heterogeneous samples. The pooling of heterogeneous samples efficiently identifies transcripts that are differentially expressed in a high proportion of the samples that comprise two groups [10]. We sought to further improve ADE methodology by accelerating prostate cancer biomarker discovery in clinically relevant samples. Although the gold standard of prostate cancer diagnosis is a prostate biopsy, this procedure is painful, invasive and subject to possible complications of bleeding and infection. Therefore, biomarkers that can be identified in samples such as blood are desirable. Since not all tumor-associated biomarkers will be detectable in blood, starting the discovery process in tumor tissue will later require the further expenditure of time and resources for validation in blood samples. In addition, blood-based biomarkers that represent a host response to tumor would not be identified if biomarker discovery is initiated in tumor samples. Special blood collection media allows the efficient recovery of undegraded RNA from whole blood, even after prolonged incubation at room temperature, making this a practical choice for clinical application. We thus decided to start the biomarker discovery process using whole blood samples.

Although ADE has typically been used to evaluate transcripts that are differentially expressed in tissue (hence, the terminology “averaged differential expression”), we adapted this technique to identify RNA transcripts that are present at different levels in whole blood RNA from two groups of patients. These transcripts are not necessarily differentially expressed in cells that are present in the blood, or even in tumor compared to normal tissue, but their presence in the blood can be used to differentiate between these two groups. In this study, we describe the use of ADE to identify an RNA transcript, RNF19A (Gene ID: 25897), that is present at higher levels in the blood of prostate cancer patients than in healthy controls. These results establish proof-of-principle that averaged differential expression (ADE) methodology can be used for the discovery of RNA markers in body fluids of men with prostate cancer and support the study of RNF19A as a potential prostate cancer biomarker.

## Methods

### Ethics Statement

All research involving human participants was approved by the Henry Ford Health System (HFHS) Institutional Review Board. Written informed consent was obtained for all participants and all clinical investigation was conducted according to the principles expressed in the Declaration of Helsinki.

### Patients and Blood Collection

Prostate cancer patients were identified through the Department of Urology at the Henry Ford Health System (HFHS). Healthy controls were recruited from the general population and HFHS Internal Medicine clinics. Whole blood (2.5 ml) was collected in PAXgene Blood RNA tubes (Qiagen, Valencia, CA), allowing for transport at room temperature without RNA degradation. Samples were frozen at −80°C until extraction of RNA was performed.

### RNA Isolation

Blood in PAXgene Blood RNA tubes was centrifuged. The pellet was washed with RNase-free water and resuspended in Trizol reagent (Invitrogen, Carlsbad, CA). RNA was extracted according to the manufacturer’s protocols. The isolated RNA was treated with RNase-free DNase (Invitrogen, Carlsbad, CA) and the concentration of total RNA was determined using a Qubit Fluorometer (Invitrogen, Carlsbad, CA).

### Averaged Differential Expression (ADE)

Total RNA from the blood of 10 patients and 10 healthy men were pooled separately, reverse transcribed, and subjected to ADE as described by Bai et al [10]. Anchor and arbitrary primers used for PCR were H-T11A and H-AP17 (GenHunter Corporation, Nashville, TN). PCR reactions were carried out in duplicate. PCR products were electrophoresed and bands detected by autoradiography. Bands that differed in intensity between prostate cancer patients and healthy men were cut from the gel, re-amplified using the same anchor and arbitrary primers H-T11A and H-AP17, and directly sequenced.

### qRT-PCR

RNA was reverse transcribed using random hexamer primers and Transcriptor Reverse Transcriptase (Roche Applied Science, Indianapolis, IN) according to the manufacturer’s protocol. After reverse transcription, RNA was subjected to qPCR on an Applied Biosystems 7500 Fast Real-Time PCR System (Applied Biosystems, Foster City, CA) using sequence-specific primers for RNF19A (Assay Id: Hs00968447_m1) and 18S RNA (Assay Id: Hs03928985_g1). The cycle number at which the reaction crossed a threshold fluorescence (C_T_) was determined for each transcript, and the level of each test gene relative to 18S RNA (reference transcript) was determined using the equation 2^−ΔC^_T_ where ΔC_T_  =  C_T,test_ – C_T,ref_ [14].

### Statistical Methods

Means were compared using a t-test. The equality of the group variances was tested, and the Satterthwaite p-value was reported for distributions with unequal variances. The Pearson correlation coefficient was used to evaluate linear correlation. A receiver operator curve (ROC) was generated by plotting the sensitivity against the false positive rate (1 − specificity) as the discrimination of the test was varied. Area under the curve (AUC) was calculated. All statistical computations were done in SAS.

## Results and Discussion

**Figure 1:**
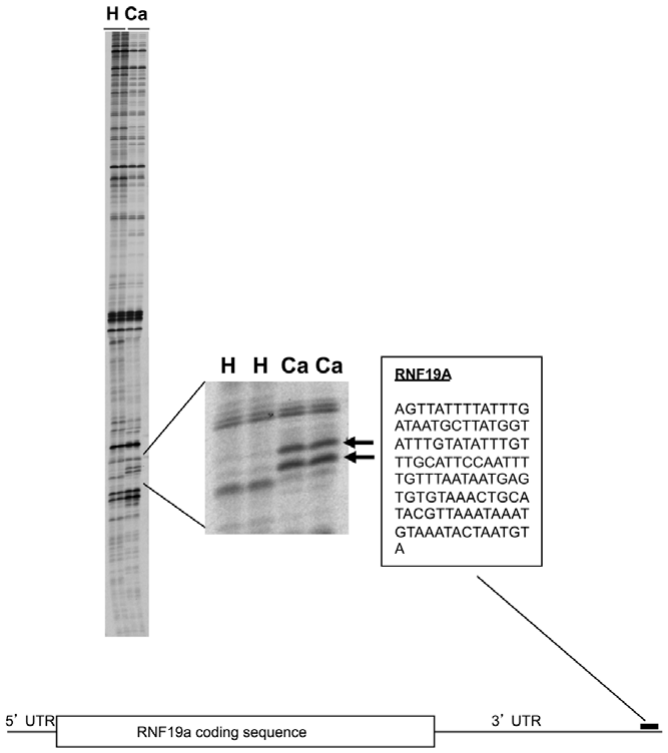
ADE analysis of whole blood RNA from prostate cancer patients vs. healthy men. ADE analysis identified two RNA transcripts with levels significantly higher in whole blood RNA from prostate cancer patients (Ca) than in healthy men (H). Nucleotide sequence analysis revealed that these two transcripts shared a common sequence. This sequence had 100% homology to the nucleotide sequence in the E3-ubiquitin ligase Ring Finger Protein 19a (RNF19A) transcript and is located in the 3′ untranslated region (UTR).

Blood samples were obtained from healthy male controls and men with localized prostate cancer prior to definitive therapy. After RNA extraction, 20 ng total RNA from each of the 10 prostate cancer patients or from each of the 10 healthy men were combined to form two separate pools of RNA (prostate cancer vs. healthy) that were then analyzed by ADE as described by Bai et al [10]. PCR amplification using an anchor and arbitrary primer set was performed in duplicate and the reaction products were subjected to gel electrophoresis (Fig. 1).

As shown in Fig. 1, ADE analysis identified two RNA transcripts amplified at significantly higher levels in blood samples from prostate cancer patients (Ca) than in those from healthy men (H). Nucleotide sequence analysis (Fig. S1, supplementary data) revealed that these two transcripts shared a common sequence and that this sequence had 100% homology to the nucleotide sequence in the ring finger protein 19A (RNF19A) mRNA transcript. RNF19A, also called Dorfin, is an E3 ubiquitin ligase known to be expressed in the pathologic inclusions found in Parkinson’s disease, amyotrophic lateral sclerosis, and Lewy body dementia [15]. RNF19A ubiquitinates synphilin-1 and mutated superoxide dismutase 1 (SOD1), which are implicated in the pathogenesis of these neurodegenerative disorders [16], [17]. RNF19A has not previously been associated with cancer, which underscores the utility of differential display in identifying novel associations. However, both RNF19A and its targets (SOD1 and synphilin-1) have been implicated in cell survival and cell death pathways [18], [19], [20]. SOD1 has been specifically associated with prostate cancer [21], [22] and may be involved in the cellular response to DNA damage through its role in the oxidative stress pathway. Other E3-ubiquitin ligases, such as RNF6, are reported to regulate androgen receptor activity in prostate cancer cells [23].

**Figure 2:**
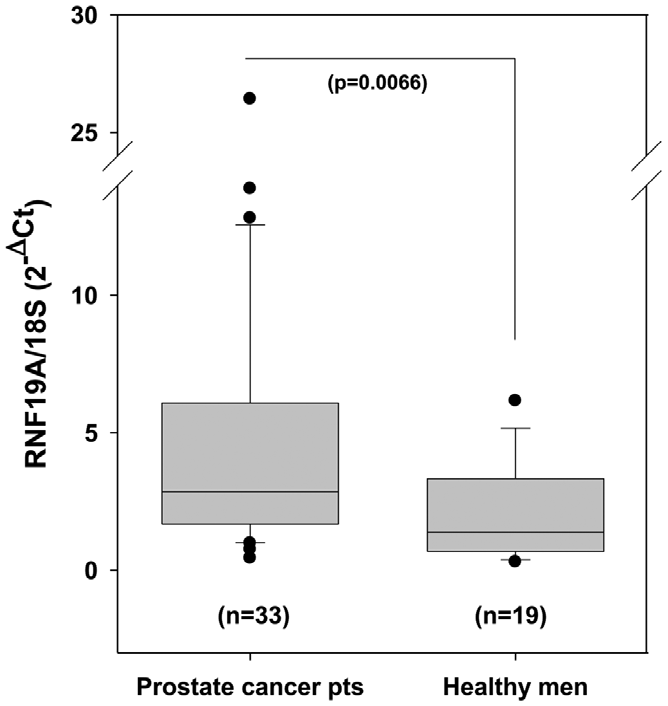
RNF19A transcript levels are higher in prostate cancer patients than in healthy men. Quantitative RT-PCR was performed on whole blood RNA samples from patients with localized prostate cancer (n = 33) as well as healthy male controls (n = 19). Levels of RNF19A transcript were normalized to 18S RNA (reference gene). Normalized results are presented in box plot format, with boxes representing the 25^th^, 50^th^, and 75^th^ percentiles and whiskers representing the 10^th^ and 90^th^ percentiles of the data. Outliers are also displayed. The difference between the means was statistically significant (p  =  0.0066).

After the identification of the RNF19A transcript as a potential biomarker distinguishing the blood of prostate cancer patients from the blood of healthy controls, qRT-PCR was used to measure the level of this transcript, relative to that of 18S RNA, in whole blood RNA samples from individual patients. Relative RNF19A levels were measured in 33 prostate cancer patients and 19 healthy male controls. These cohorts were independent of the patient groups used for the ADE discovery process. The control samples (median age 40 yrs, range 31–50 yrs) were taken from the general population, and as such, were not age-matched to prostate cancer cases. Baseline clinical data for the prostate cancer cohort are shown in Table 1. All prostate cancer cases had conventional adenocarcinoma on pathologic review and none were documented to have nodal or distant metastatic disease. Relative RNF19A levels as measured by qRT-PCR are shown in Fig. 2 (for comparison of raw C_T_ data, please see supplementary data, Table S1). The mean relative level of RNF19A was 4.79 ± 0.91 (SE) in prostate cancer patients compared to 1.96 ± 0.39 (SE) in healthy controls. This difference between the two groups was statistically significant (p = 0.0066). A similar statistically significant difference in RNF19A levels between prostate cancer patients and healthy controls was obtained when levels were assessed using semi-quantitative RT-PCR (Fig. S2, supplementary data).

**Table 1:** Clinical characteristics of the validation prostate cancer cohort.

		Median	Range
Age (yrs)		66	53–82
PSA (ng/mL)		6.4	1.3–33.4
			
		Number	Percent
Gleason			
	6	8	24%
	7	18	55%
	8–10	7	21%
T-stage			
	cT1a	1	3%
	cT1c	30	91%
	cT2a	1	3%
	cT3a	1	3%

Characteristics of the 33 patients included in the prostate cancer validation cohort are shown. Age and baseline PSA were abstracted from the time of diagnosis. Gleason score is as recorded by the clinical genitourinary pathologist. Clinical staging of the primary tumor is per the 7^th^ edition of the American Joint Committee on Cancer (AJCC) staging manual.

**Figure 3:**
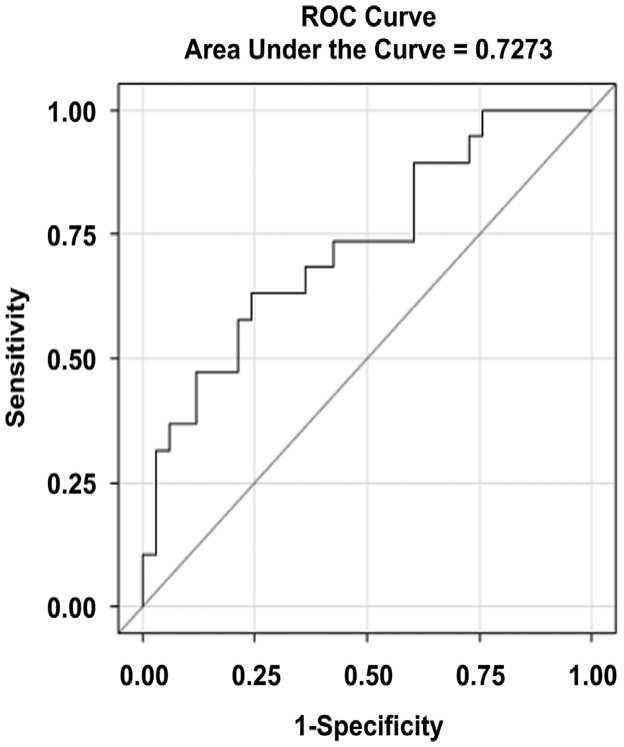
ROC curve evaluating the accuracy of RNF19A as a diagnostic test. A receiver-operating curve (ROC) was generated as a preliminary estimate of the accuracy of relative levels of RNF19A in classifying patients with cancer or healthy controls in our cohort of patients. The true positive rate (sensitivity) was plotted against the false positive rate (1-specificity). The area under the curve was calculated as 0.7273.

A receiver-operating curve for RNF19A was generated as an overall estimate of the sensitivity and specificity of this blood biomarker for distinguishing prostate cancer cases from controls (Fig. 3). The AUC for the model was 0.7273, where an AUC of 1 corresponds to a diagnostic test with perfect (100%) specificity and sensitivity. As a point of reference, the AUC for PSA has been estimated at 0.640–0.678, while the AUC for a popular nomogram that takes into account other prostate cancer risk factors in addition to the PSA level has been estimated at 0.691 [24], [25], [26].

Although this finding is encouraging, it should be noted that using PSA to differentiate patients with prostate cancer from controls in our cohorts may have also resulted in an improved AUC given the relatively younger age of our healthy cohort. With a median age of 40, the expected PSA in our healthy cohort would be less than 1.0 ng/mL, substantially lower than the median PSA value of 6.4 ng/mL seen in our prostate cancer cohort. However, RNF19A levels did not correlate with age (Pearson coefficient of 0.09), making it unlikely that our findings are simply due to an age-related effect. Clearly, further validation needs to be performed in populations that more closely resemble the real-world setting of patients who are being referred for prostate biopsy. In addition, it remains to be determined whether RNF19A or other such biomarkers will add additional value to clinically established methods to establish prostate cancer risk such as PSA, age, race and family history. This study was not designed to address the critical question of increased detection of potentially lethal cancer while avoiding the overdiagnosis of indolent cancer. Further efforts at biomarker discovery to identify these lethal subtypes are an essential goal for the future.

In conclusion, we describe here the use of averaged differential expression to identify RNF19A as an RNA transcript that is present at significantly higher levels in the blood of prostate cancer patients compared to healthy controls. In our sample of patients, this transcript was able to distinguish prostate cancer patients from controls with an AUC of the receiver-operating curve of 0.7273. The results discussed here establish proof-of-principle that ADE methodology can be used for the discovery of RNA markers in the blood of men with prostate cancer and warrant further analysis of RNF19A as a clinically relevant biomarker for prostate cancer early detection.

## Supporting Information

Figure S1 **Chromatograms of ADE-identified transcripts.** Samples from healthy controls and patients with prostate cancer were subjected to averaged differential expression analysis. Transcripts (A and B), present at different levels in healthy controls compared to prostate cancer patients, were submitted for sequencing. Chromatograms from the sequencing reactions for both transcript A and B are shown, indicating an identical common sequence. Given the resolution of the differential display gel, the difference in length between the two bands (A and B) is no more than several nucleotides. A different annealing location for either the random primer or the polyA primer may explain this result.
(PPT)

Figure S2 **RT-PCR analysis of RNF19A in blood from prostate cancer patients and healthy men.** Levels of RNF19 were evaluated using semi-quantitative RT-PCR. RNA was reverse transcribed using random hexamers or oligo (dT) primer and Transcriptor Reverse Transcriptase (Roche Applied Science) according to the manufacturer’s protocol. Amplification of cDNA was done using sequence-specific primers of RNF19A and GAPDH genes. PCR products were run on a 2% agarose gel. Quantitation of cDNA bands on the gel was carried out by digital analysis of band intensity using an Eagle Eye II still video system with the software provided by Stratagene (La Jolla, CA). RNF19A transcript levels were significantly higher in prostate cancer patients compared to controls.
(PPT)

Table S1 Raw C_T_ data are presented for the validation cohorts of prostate cancer patients (PrCa pts) and healthy controls. Mean C_T_ values were lower in PrCa pts compared to controls (28.25 vs. 29.50, p = 0.02). Mean C_T_ values for the 18S reference gene were not statistically different between patients and controls (16.62 vs 16.67, p = 0.89). Means were compared using a t-test.
(DOC)
