# Supplementary figures and images for: Averaged Differential Expression for the Discovery of Biomarkers in the Blood of Patients with Prostate Cancer
Source: PLoS One. 2012 Apr 6;7(4):e34875. doi: 10.1371/journal.pone.0034875 (PMC3321043; doi:10.1371/journal.pone.0034875)

## Slide 1
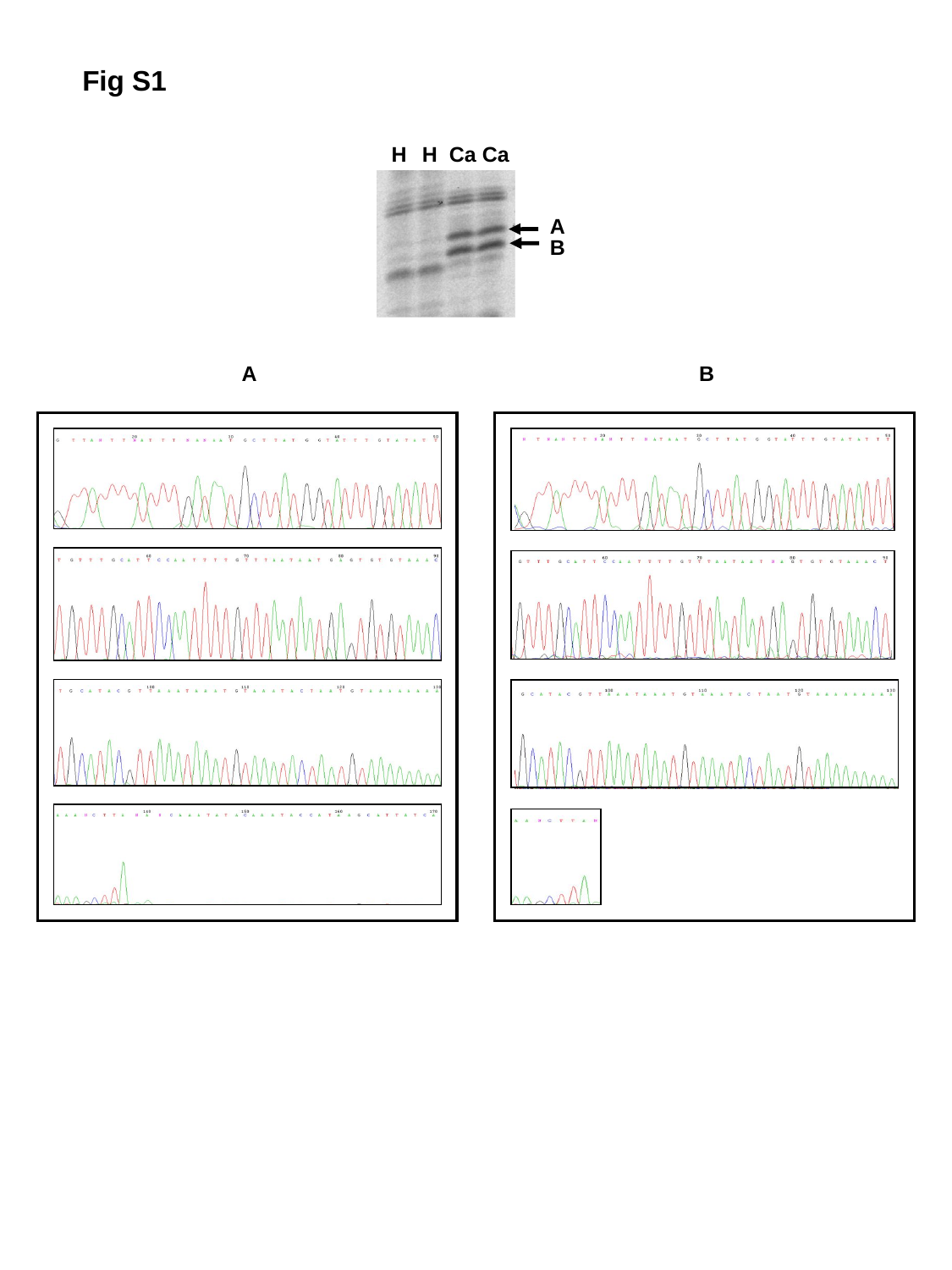

Fig S1
 H H Ca Ca
 A
 B
 A
 B

Supplement: Figure S1 — Chromatograms of ADE-identified transcripts. Samples from healthy controls and patients with prostate cancer were subjected to averaged differential expression analysis. Transcripts (A and B), present at different levels in healthy controls compared to prostate cancer patients, were submitted for sequencing. Chromatograms from the sequencing reactions for both transcript A and B are shown, indicating an identical common sequence. Given the resolution of the differential display gel, the difference in length between the two bands (A and B) is no more than several nucleotides. A different annealing location for either the random primer or the polyA primer may explain this result. (PPT) [file pone.0034875.s001.ppt]

## Slide 1
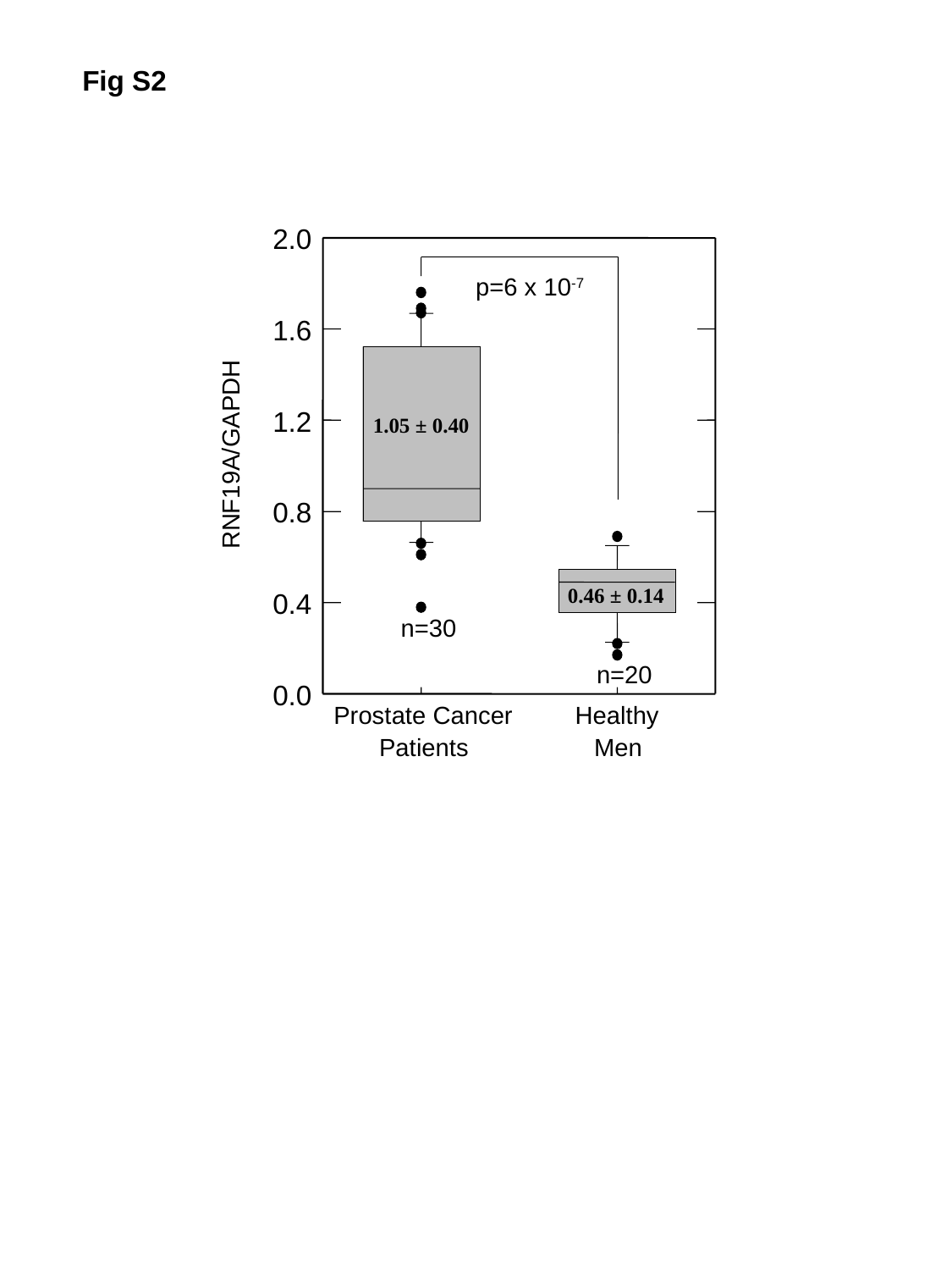

Fig S2
2.0
p=6 x 10-7
1.6
1.2
1.05 ± 0.40
RNF19A/GAPDH
0.8
0.46 ± 0.14
0.4
n=30
n=20
0.0
Prostate Cancer
Healthy
Patients
Men

Supplement: Figure S2 — RT-PCR analysis of RNF19A in blood from prostate cancer patients and healthy men. Levels of RNF19 were evaluated using semi-quantitative RT-PCR. RNA was reverse transcribed using random hexamers or oligo (dT) primer and Transcriptor Reverse Transcriptase (Roche Applied Science) according to the manufacturer’s protocol. Amplification of cDNA was done using sequence-specific primers of RNF19A and GAPDH genes. PCR products were run on a 2% agarose gel. Quantitation of cDNA bands on the gel was carried out by digital analysis of band intensity using an Eagle Eye II still video system with the software provided by Stratagene (La Jolla, CA). RNF19A transcript levels were significantly higher in prostate cancer patients compared to controls. (PPT) [file pone.0034875.s002.ppt]
