# Supplementary material for: Averaged Differential Expression for the Discovery of Biomarkers in the Blood of Patients with Prostate Cancer
Source: PLoS One. 2012 Apr 6;7(4):e34875. doi: 10.1371/journal.pone.0034875 (PMC3321043; doi:10.1371/journal.pone.0034875)
Supplement: Table S1 — Raw CT data are presented for the validation cohorts of prostate cancer patients (PrCa pts) and healthy controls. Mean CT values were lower in PrCa pts compared to controls (28.25 vs. 29.50, p = 0.02). Mean CT values for the 18S reference gene were not statistically different between patients and controls (16.62 vs 16.67, p = 0.89). Means were compared using a t-test. (DOC) [file pone.0034875.s003.doc]

# Table S1. Comparison of C_T_ data between prostate cancer patients and healthy controls.

|  |  | PrCa pts | Controls | p-value |
| --- | --- | --- | --- | --- |
| 18S (C_T_) |  |  |  |  |
|  | Mean | 16.62 | 16.67 | p=0.89 |
|  | Range | 13.53-18.25 | 13.58-19.04 |  |
|  | SD* | 0.90 | 1.92 |  |
|  |  |  |  |  |
| RNF19A (C_T_) |  |  |  |  |
|  | Mean | 28.25 | 29.50 | p=0.02 |
|  | Range | 26.27-30.89 | 24.24-32.74 |  |
|  | SD* | 1.236 | 2.56 |  |

*SD = standard deviation

Raw C_T_ data are presented for the validation cohorts of prostate cancer patients (PrCa pts) and healthy controls. Mean C_T_ values were lower in PrCa pts compared to controls (28.25 vs. 29.50, p=0.02). Mean C_T_ values for the 18S reference gene were not statistically different between patients and controls (16.62 vs 16.67, p=0.89). Means were compared using a t-test.
